# An Acoustic Underwater Glider for the Real-Time Transmission of Observation Data via an Underwater Acoustic Communication Modem

**DOI:** 10.3390/s25030849

**Published:** 2025-01-30

**Authors:** Sichen Zou, Qindong Sun

**Affiliations:** 1 National Key Laboratory of Underwater Acoustic Technology, Harbin Engineering University, Harbin 150001, China; zousichen_1992@163.com; 2 Qingdao Innovation and Development Center of Harbin Engineering University, Qingdao 266400, China; 3 Laoshan Laboratory, Qingdao 266237, China; 4 School of Mechanical Engineering, Tianjin University, Tianjin 300350, China; 5 Naval Submarine Academy, Qingdao 266199, China; 6 Qingdao Institute of Collaborative Innovation, Qingdao 266071, China

**Keywords:** acoustic underwater glider, underwater acoustic communication, underwater acoustic modem

## Abstract

This paper introduces the development of an acoustic underwater glider integrated with an underwater acoustic modem designed to enable the real-time transmission of ocean observation data. The glider features three sequentially connected, independent compartments and is capable of operating at depths exceeding 1000 m. To ensure stable communication, two acoustic transducers are mounted at the rear of the glider and optimized to maintain a consistent energy radiation angle despite variations in the glider’s attitude. The acoustic modem, housed within one of the compartments, operates with a standby power consumption as low as 5 mW, significantly enhancing the overall energy efficiency of the system. To address the glider’s motion dynamics and the unique characteristics of the underwater acoustic channel, a multi-carrier frequency shift keying-based underwater acoustic communication scheme combined with a Stop-and-Wait Automatic Repeat Request protocol was designed and implemented. The system’s performance and reliability were validated through sea trials conducted in the South China Sea. The results demonstrated that the glider achieved reliable underwater acoustic communication over distances of up to 5 km. This research highlights the potential of the acoustic underwater glider for applications such as underwater acoustic measurements and distributed networking collaboration. The system holds significant promise for advancing underwater acoustic communication and ocean observation technologies.

## 1. Introduction

The underwater glider represents a common type of autonomous underwater vehicle, primarily employing buoyancy to facilitate propulsion and enable vertical movement within the ocean [1]. During the initial descent phase, internal buoyancy units must be adjusted to render the glider heavier than the surrounding buoyancy, thus enabling its descent. Once the glider reaches the predetermined depth, buoyancy units are readjusted to alter its displacement volume, ensuring that its buoyancy exceeds its weight, consequently causing it to ascend. This mechanism, coupled with wings, facilitates the conversion of vertical motion into horizontal motion, thereby enabling the glider to execute a zigzag gliding motion along a longitudinal plane [2].

Traditionally, underwater gliders have operated by alternating between floating and diving to collect observational and detection data, periodically surfacing to transmit the data via satellite communication [3,4]. However, this operational mode compromises the timeliness of marine data collection, potentially resulting in missed opportunities for decision-making and limiting the ability to meet short-term data acquisition demands. To overcome these limitations and improve the efficiency of marine exploration and target detection, increasing attention has been directed toward integrating underwater acoustic (UWA) communication technology into underwater gliders and other submersible vehicles [5,6,7,8,9,10].

Underwater acoustic (UWA) communication is a critical technology for establishing information links among underwater vehicles. However, the UWA channel poses significant challenges, characterized by large delay spreads and pronounced Doppler effects [11,12]. Additionally, the diverse operational modes and navigation states of various types of underwater vehicles further complicate the implementation of reliable UWA communication [13,14,15,16,17]. For underwater vehicles, particularly gliders, diving and floating maneuvers often result in significant rolling and pitching, highlighting the absence of an optimal communication protocol suited to such dynamic environments.

Currently, the most widely used modems for underwater vehicles primarily adopt Frequency Shift Keying and Orthogonal Frequency Division Multiplexing (OFDM) modulation techniques [18,19]. OFDM is effective for mitigating multipath interference [20] but is highly sensitive to Doppler shifts and requires a high signal-to-noise ratio (SNR) for reliable reception [21]. In contrast, FSK offers stable and reliable communication, albeit at lower transmission rates [22,23]. The adoption of a multi-carrier frequency shift keying (MFSK) communication scheme enables the accommodation of diverse operational parameters, making it particularly well suited for underwater vehicle platforms.

This paper introduces a type of acoustic underwater glider equipped with a UWA communication system designed for observation, collecting data, and conducting real-time transmission. The UWA communication device is housed within the glider’s cabin, with two UWA transducers mounted at the stern of the cabin. Additionally, a communication scheme based on multi-carrier frequency shift keying has been developed to align with the motion characteristics of the underwater glider and the unique features of UWA channels. Finally, a sea trial was conducted in the South China Sea to verify the performance of the acoustic underwater glider in UWA communication, effectively expanding the range of capabilities for acoustic gliders and enhancing their surveillance capabilities.

## 2. Glider Design

### 2.1. Structural Design of the Acoustic Glider

**Figure 1:**
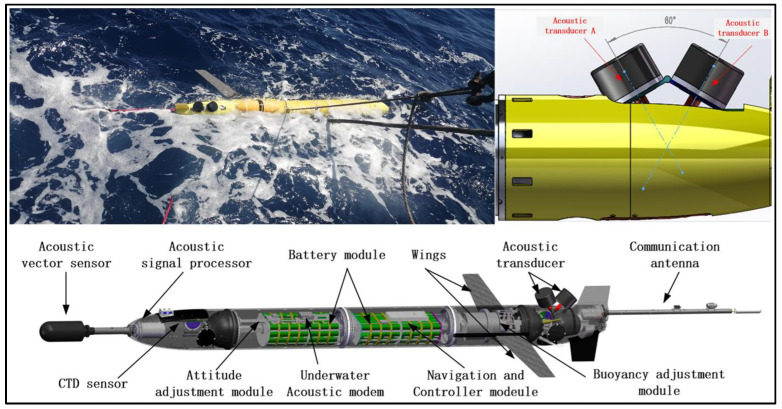
Exploded view of the acoustic sea glider.

The acoustic underwater glider measures 3.2 m in length and 0.25 m in diameter. It maintains an average speed of two knots, with a maximum diving depth of 1000 m. The glider comprises three independent passenger cabins, as depicted in Figure 1.

The forward cabin, positioned at the front of the glider, primarily serves to accommodate observation and detection sensors, including acoustic modules and CTD sensors. The acoustic modules encompass an acoustic vector sensor and a signal treatment cabin. The dimensions of the vector sensor are ∅66 × 78 mm. Test results indicate that the sensitivity of the hydrophone is −191.5 dB (0 dB re 1 V/μPa), and the sensitivities of the triaxial piezoelectric accelerometer are 2.85 V/g, with equivalent pressure sensitivities of −179 dB (0 dB re 1 V/μPa at 1 kHz), and its operational bandwidth extends from 10 Hz to 3 kHz. The acoustic signal processor is situated within the front cabin to capture and store signals emitted by the vector sensor.

The mid-cabin serves as a pressurized compartment divided into three sections. The initial section houses the signal processor for UWA communication, a posture adjustment module, a set of batteries, and a driving mechanism. Within the second section, a navigation control module and a series of spare battery packs are positioned. Notably, the navigation control module necessitates data interaction with the signal processor of UWA communication. The third section accommodates an oil pump, which regulates buoyancy by injecting or extracting oil from the oil bag. Additionally, carbon-fiber wings are affixed to each side of the third section to facilitate pitch angle adjustment.

The stern cabin is immersed in seawater, while the gravity perpendicular of two UWA transducers is tilted at an angle of 30°. These transducers are connected to the signal processor of UWA communication through the cabin’s electric cable, facilitating the transmission and reception of signals of underwater sounds. The two UWA transducers can operate alternately underwater, ensuring that one remains in a vertically upward state to maintain consistent energy transmission range and angle, even as the underwater glider’s posture changes. The oil bag serves as a component of the buoyancy adjustment module, providing the necessary buoyancy for the glider to float and dive. Additionally, propellers and satellite communication antennae are positioned at the rear end of the stern cabin.

### 2.2. Modem Design

Underwater gliders utilize half-duplex communication to enable the delivery of shore-based instructions and the real-time transmission of observational data. This requires the continuous reception and processing of UWA signals, which demands a persistent data reception and communication protocol, placing considerable strain on the glider’s battery resources. Given that battery replacement for underwater gliders is both logistically challenging and cost-prohibitive, minimizing energy consumption is critical to extending their operational lifespan. Furthermore, the limited internal space within the glider presents additional challenges for the design and integration of the signal processor.

**Figure 2:**
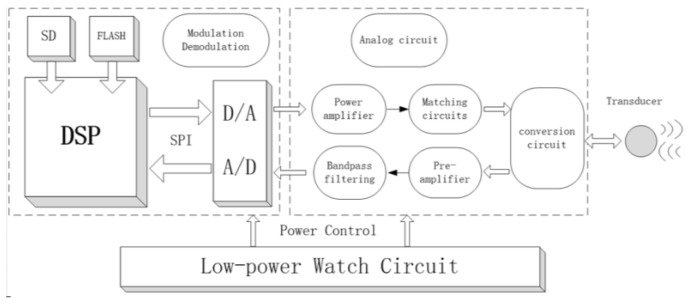
Low-power signal processor.

Considering both energy consumption and spatial constraints, the signal processor is designed with compact dimensions of 10.5 mm × 5.5 mm × 5.5 mm, integrating three core functionalities: data reception and modulation, high-efficiency signal transmission, and low-power signal processing, as shown in Figure 2.

The modulation of received data primarily involves functions such as amplification, filtering, and gain control. The high-efficiency signal transmission module integrates a D-class power amplifier and a matching network, enabling UWA signal transmission with a sound source level exceeding 186 dB. The low-power signal processing module incorporates a low-power watch circuit and a high-speed DSP processor, which are responsible for low-power signal detection and communication positioning algorithms, respectively.

The low-power watch circuit manages the system’s power supply, remaining active during idle states to monitor external signals. Upon detecting a wake-up signal, it activates the working circuit, initiating system wake-up and transitioning the system to operational mode. This mechanism ensures reliable signal reception and system activation while significantly reducing energy consumption during periods of signal absence, thereby optimizing energy usage and enhancing power supply efficiency. The electronic module interfaces with the glider through power cables and an RS-232 serial port connection.

**Figure 3:**
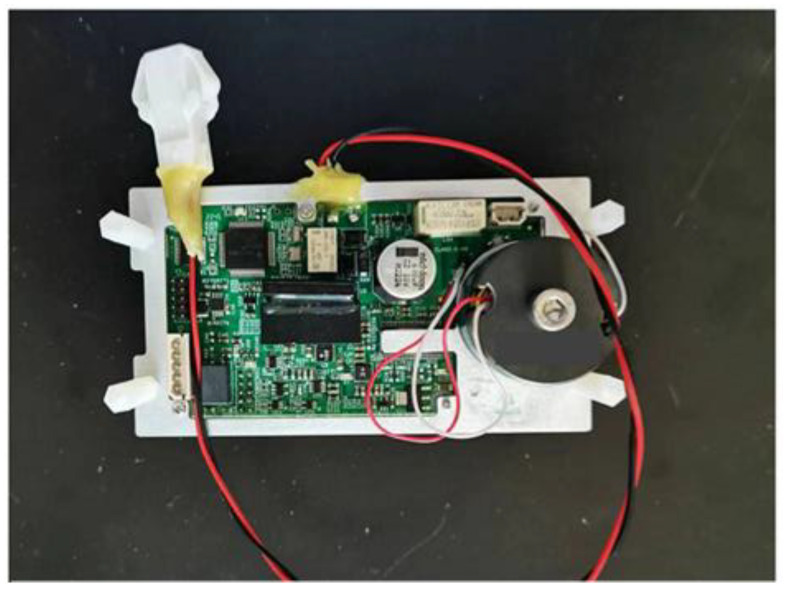
Modem hardware.

The low-power watch circuit design uses the MSP430FR5994 microcontroller as the core processor, which features power consumption of only 120 µA/MHz in operational mode. The operating frequency of the low-power watch circuit in this study is 8 MHz, resulting in a working current of 8 MHz * 120 µA/MHz = 0.96 mA. Including the power consumption of other hardware components, the total power consumption is approximately 2 mA. The microcontroller operates at a voltage of 2.5 V, leading to a total power consumption of 5 mW. The modem hardware is shown in Figure 3.

**Figure 4:**
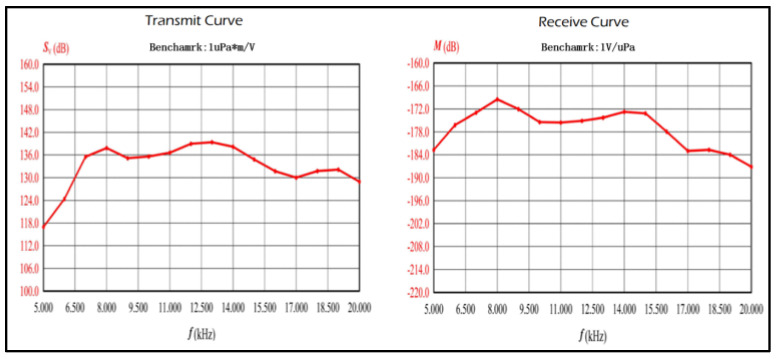
The frequency response of the UWA transducer.

The UWA transducer adopts a ceramic-ring, semi-space, omnidirectional, oil-filled design, offering superior performance compared to porcelain-based energy transducers in terms of directivity, transmitting voltage response, and maximum sound source level. Its operational frequency ranges from 8 kHz to 16 kHz, with a transmitting voltage response exceeding 136 dB, receiving sensitivity surpassing −178 dB, and a maximum emission sound source level of 195 dB. The frequency response characteristics of the UWA transducer are shown in Figure 4.

### 2.3. Dynamic Modeling and Simulation

**Figure 5:**
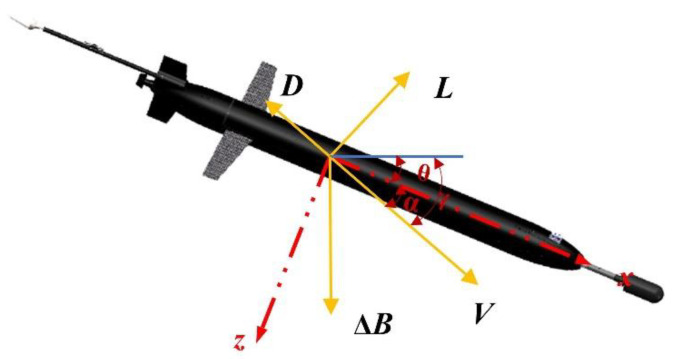
Force analysis and angle relationship of the glider.

After the design of the glider, its dynamic modeling can be established, and the motion process of the glider can be approximated as a steady-state gliding motion in the longitudinal plane of the profile. Force analysis and angle relationship of the glider are presented in Figure 5.

The force and angle of the glider satisfy the following relationship [24]:

(1) $$\left\{\begin{array}{c} \begin{array}{c} \gamma =\theta -\alpha \\ ∆B\cos\gamma =L \end{array}\\ ∆B\sin\gamma =D \end{array}\right.$$

where α, θ, and γ denote the attack angle, pitch angle, and gliding angle, respectively, ∆B, L, and D represent net buoyancy, lift, and drag.

**Figure 6:**
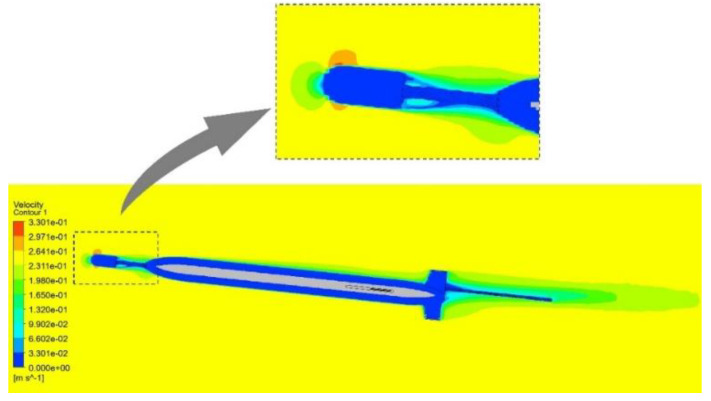
Hydrodynamic simulation of the glider.

The lift and drag can be expressed as follows [24]:

(2) $$\left\{\begin{array}{c} L=0.5\rho_{w}AV^{2}C_{L}\\ D=0.5\rho_{w}AV^{2}C_{D} \end{array}\right.$$

where $\rho_{w}$ is the density of seawater, A represents the cross-sectional area in the *x*-axis direction, V denotes the gliding velocity of the glider, and $C_{L}=a\alpha$ and $C_{D}=b+c\alpha^{2}$ represent the lift coefficient and drag coefficient, respectively, which can be obtained through hydrodynamic shape simulation. In this study, hydrodynamic shape simulation is conducted with Computational Fluid Dynamics (CFD), as shown in Figure 6.

Taking underwater gliding as an example, the net buoyancy ∆B during the process can be expressed as

(3) $$∆B\left(h\right)=\rho_{w}gV_{g}\left(1-kP\left(h\right)+\kappa \Delta T\left(h\right)\right)-M_{g}g-\rho_{w}gV_{in}$$

where $V_{g}$ and $M_{g}$ represent the volume and mass of the glider, P represents the pressure of seawater, ΔT represents the variation of the temperature of seawater, k denotes the compression coefficient, κ is the coefficient of thermal expansion, and $V_{in}$ represents the volume of oil return during the dive phase.

Gliding velocity $V\left(h\right)$ and $\gamma \left(h\right)$ gliding angle with depth can be calculated by solving systems of nonlinear equations:

(4) $$\left\{\begin{array}{c} \cot\left(\theta -\alpha \right)=C_{L}/C_{D}\\ \Delta B\cos\left(\theta -\alpha \right)=0.5\rho_{w}AV^{2}C_{L} \end{array}\right.$$

Then, gliding time $T_{0}^{H}$ and gliding range $R_{0}^{H}$ of the diving phase can be obtained:

(5) $$T_{0}^{H}=\frac{H^{2}}{\int_{0}^{H}V\left(h\right)\sin\left(\theta -\alpha \left(h\right)\right)dh}$$

(6) $$R_{0}^{H}=\left(\frac{\int_{0}^{H}V\left(h\right)\sin\left(\theta -\alpha \left(h\right)\right)dh}{H}\right)\times T_{0}^{H}$$

Similarly, the gliding time and range of the climbing phase can be calculated; then, gliding time T and gliding range R can be obtained:

(7) $$\left\{\begin{array}{c} T=T_{0}^{H}+T_{H}^{0}\\ R=R_{0}^{H}+R_{H}^{0} \end{array}\right.$$

**Figure 7:**
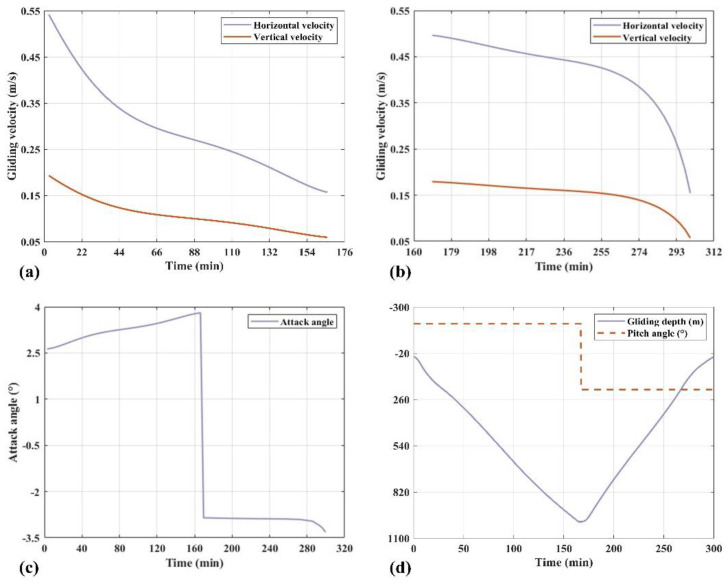
The results of dynamic simulations. (**a**) The gliding velocity of the diving phase. (**b**) The gliding velocity of the climbing phase. (**c**) The attack angle of the gliding phase. (**d**) The pitch angle and gliding trajectory of the gliding phase.

Once the dynamic model has been established, the motion of the glider can be simulated. By inputting motion parameters, numerical simulation and analysis of the motion process are conducted in the MATLAB environment. Figure 7 presents the results of dynamic simulations.

The results of dynamic simulations indicate that the designed acoustic glider is capable of diving to a depth of 1000 m.

## 3. Communication Scheme

### 3.1. The Transmitted Signal

During the underwater glider’s operation, its trajectory alternates periodically between shallow and deep waters. This periodic motion pattern substantially influences the performance of underwater acoustic communication, particularly in scenarios where acoustic channel conditions differ significantly between shallow and deep water environments. Additionally, underwater acoustic channels are inherently affected by severe multipath interference and Doppler shifts. In dynamic communication scenarios involving mobile platforms such as underwater gliders, the Doppler effect becomes even more pronounced, further degrading signal quality.

**Figure 8:**
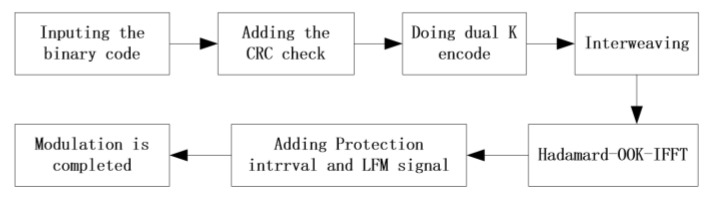
Process flow chart of MFSK modulation.

To adapt to the complex conditions of underwater acoustic channels, the underwater glider is better suited to low-speed and robust acoustic communication technologies. To balance communication speed and reliability, the acoustic glider employs a multi-carrier frequency shift keying (MFSK) acoustic communication technology, which offers strong anti-interference capabilities and adaptability to complex underwater acoustic channels. The frame structure for data frame modulation is shown in Figure 8.

**Figure 9:**
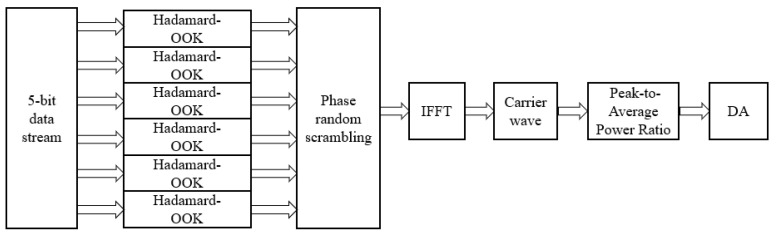
Hadamard-OOK-IFFT modulation.

The modulation process utilizes Hadamard codes, which are characterized by an equal number of 0 s and 1 s. This property enhances the coding gain under the same SNR, thereby improving the robustness of the underwater glider’s communication performance. Following channel encoding, the input bitstream is mapped onto multiple subcarriers within a specified bandwidth using inverse fast Fourier transform (IFFT). The modulation process of the Hadamard code is shown in Figure 9.

Furthermore, the effective frequency points within the bandwidth are spaced sufficiently apart, providing resilience to Doppler frequency shifts. This design achieves an optimal balance between communication speed and stability, ensuring reliable performance in dynamic underwater environments.

Consider one cyclic prefix (CP) MFSK block from the transmission illustrated, where each MFSK block consists of N subcarriers. The subcarrier spacing is defined as Δf=B/N, where B denotes the system bandwidth. The frequency associated with the kth subcarrier is given by

(8) $$f_{n}=f_{c}+n\Delta f\;,\;n=-N/2,\ldots ,N/2-1$$

where $f_{c}$ represents the carrier frequency. The period of an MFSK block is defined as $T=T_{d}+T_{cp}$, where $T_{d}=1/\Delta f$ is the duration of the MFSK symbol, and $T_{cp}$ represents the length of the cyclic prefix. The transmitted signal of the CP-MFSK block can be expressed as

(9) $$x\left(t\right)=2Re\left\{\sum_{n=-N/2}^{N/2-1}x\left[n\right]e^{j2\pi f_{n}t}w\left(t\right)\right\},t\in \;\left[-T_{cp},T_{d}\right]$$

In the above Equation, $x\left[n\right]$ represents the symbol transmitted on the nth subcarrier in the block while $w\left(t\right)$ refers to the pulse-shaping filter:

(10) $$w\left(t\right)=\left\{\begin{array}{c} 1,t\in [-T_{cp},T_{d}]\\ 0,\;\;otherwise. \end{array}\right.$$

Linear Frequency Modulation (LFM) signals are selected as synchronization signals due to their excellent autocorrelation properties, enabling high-precision time synchronization. Additionally, LFM signals are insensitive to Doppler effects, making them well suited for the dynamic communication scenarios of underwater gliders.

### 3.2. Receiver Processing

**Figure 10:**
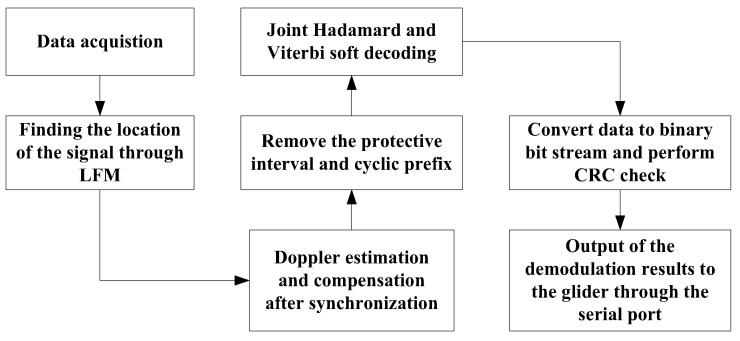
Process flow chart of MFSK demodulation.

In the communication system of the underwater glider, the signal processing workflow at the receiver plays a critical role, particularly in complex underwater acoustic channel environments. The system must address challenges such as multipath interference and Doppler frequency shifts to ensure accurate demodulation and decoding of received signals. To achieve this, the receiver’s signal processing workflow includes synchronization detection, Doppler frequency shift estimation and compensation, demodulation, and joint soft-decision decoding. The full demodulation process is depicted in Figure 10.

At the receiver, the local synchronization signal is correlated with the received signal using a matched filter. A prominent correlation peak emerges in the matched filter’s output, which the system compares against a predefined threshold to determine whether synchronization has been successfully detected.

In this study, two continuous wave (CW) signals are employed for Doppler estimation. Assuming the transmitted single-frequency signal has a frequency $f_{0}$, the relative velocity between the transmitter and receiver is v, the speed of sound is c, and the angle between the direction of sound wave propagation and the relative motion velocity is θ, the Doppler frequency shift $∆f_{d}$ is given by

(11) $$∆f_{d}=\frac{v}{c}f_{0}\cos\theta$$

Define the Doppler frequency offset factor as

(12) $$a=\frac{v}{c}\cos\theta =\frac{∆f_{d}}{f_{0}}$$

At the receiver, the CW signals, after experiencing Doppler frequency shifts, are processed using a Fast Fourier Transform (FFT) to determine their shifted frequencies. The Doppler frequency shift factors for the two CW signals are then calculated, and their average is used as the system’s overall Doppler shift factor.

Using the Doppler shift factor, the system calculates the Doppler-expanded cyclic prefix length, the symbol duration of a single MFSK signal, and the carrier signal length and determines the starting position of the signal. During signal processing after Doppler frequency shift compensation, the unquantized frequency point energy information output by the receiver’s demodulator is directly fed into the joint decoder for soft-decision decoding.

The joint decoder utilizes a soft-decision mechanism that integrates Hadamard coding and Viterbi decoding during the demodulation phase, significantly enhancing the robustness and reliability of the decoding process.

Following joint soft-decision decoding, the receiver outputs the final decoded bitstream, serving as a reliable input for the upper-layer data processing module. The entire demodulation workflow is designed with a core focus on ensuring the accuracy and robustness of data transmission, effectively addressing the challenges posed by dynamic and complex underwater acoustic channel environments.

### 3.3. Communication Protocol

Due to the periodic variation in the profiling glide depth of underwater gliders, different operating waters can exhibit significantly distinct underwater acoustic channel conditions. This dynamic and complex channel environment presents substantial challenges to the reliability and efficiency of underwater communication. As a result, establishing a feedback mechanism from the receiver to the transmitter to monitor communication outcomes becomes particularly critical.

To address this challenge, this study designed and implemented a communication mechanism based on the Stop-and-Wait Automatic Repeat Request (ARQ) protocol. In this mechanism, the transmitter sends one data packet at a time and waits for an acknowledgment (ACK) signal from the receiver. Upon receiving a correct ACK, the transmitter proceeds to send the next data packet. Conversely, if no ACK is received within the specified waiting time or if a corrupted ACK is detected, the transmitter retransmits the current data packet.

**Figure 11:**
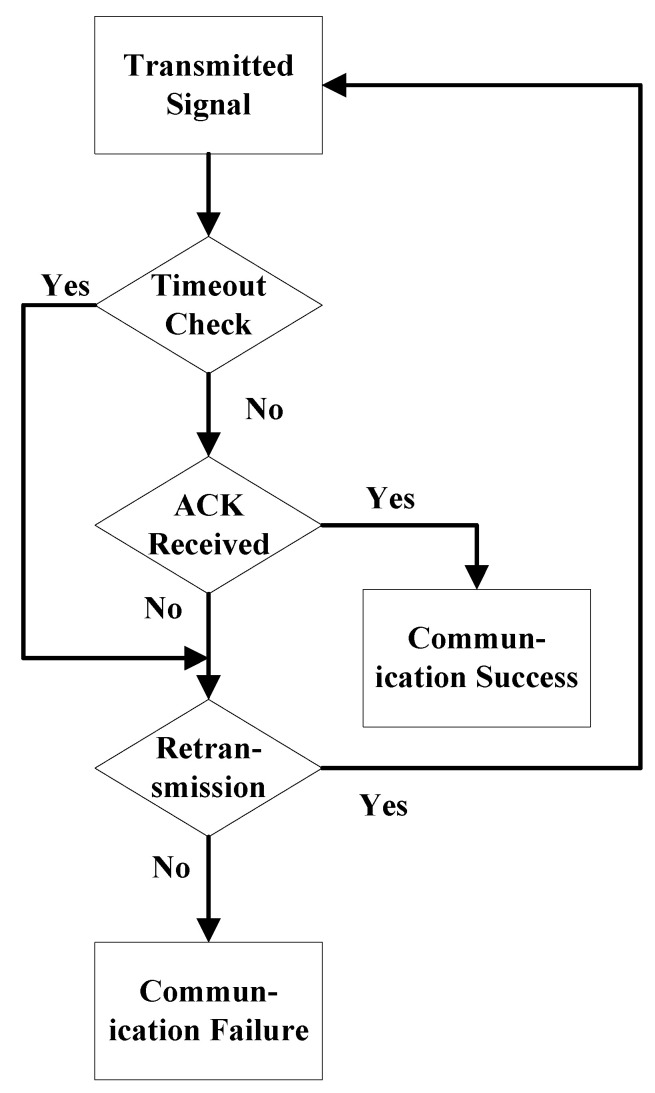
Stop-and-Wait ARQ protocol.

This feedback-based mechanism ensures reliable data transmission by minimizing packet loss and transmission errors through retransmission, even in dynamic and challenging underwater acoustic channel environments. The workflow of the Stop-and-Wait ARQ protocol is illustrated in Figure 11.

The workflow can be divided into the following steps: First, the transmitter sends a data packet to the receiver and starts a timer. Next, the receiver verifies the received data packet and responds with either an ACK or a negative acknowledgment signal. If the transmitter receives a correct ACK signal within the timer’s specified duration, it proceeds to send the next data packet. Conversely, if no ACK is received or an incorrect feedback signal is detected, the transmitter retransmits the current data packet. This process repeats until all data packets are successfully transmitted.

Compared to other more complex ARQ protocols, the Stop-and-Wait ARQ protocol has a lower channel utilization rate due to its single-packet transmission approach. However, it is straightforward to implement and particularly suitable for scenarios such as underwater gliders, which demand high communication reliability and operate in dynamic underwater acoustic environments. Moreover, this protocol helps mitigate severe data loss caused by channel fluctuations, providing a robust technical foundation for the stable communication of underwater gliders.

**Figure 12:**
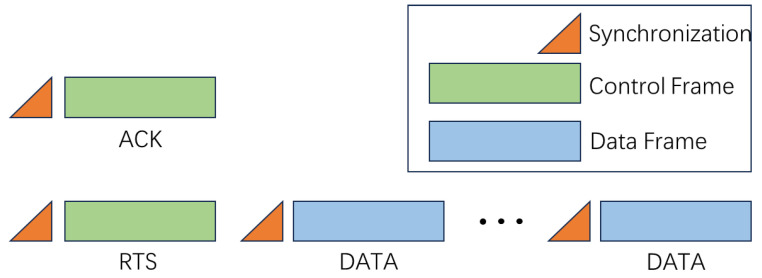
Control frame and data frame.

The data packets in the Stop-and-Wait ARQ protocol consist of a Request to Send (RTS) control frame and multiple data frames, while the acknowledgment packet comprises an ACK control frame, as shown in Figure 12.

The design of data packets and acknowledgment packets is not only crucial for communication efficiency and reliability but also plays a pivotal role in signal interaction within complex underwater acoustic channels. The RTS control frame in the data packet and the ACK control frame in the acknowledgment packet work together to establish the system’s communication feedback mechanism.

The RTS control frame carries essential information from the transmitter, ensuring the smooth transmission of data packets. Meanwhile, the ACK control frame provides feedback on verification results and channel state information, enabling the transmitter to dynamically adjust its communication strategy. By leveraging the coordinated operation of these components, the system adapts to the complex and variable underwater acoustic channel environment, achieving efficient and reliable underwater communication.

## 4. Sea Trial

To evaluate the communication performance of the acoustic underwater glider, a surface wave-energy glider integrated with a UWA communication system was employed as the surface relay. A one-to-one communication test was subsequently conducted in the South China Sea, with the trial area having a depth of approximately 1800 m. The system parameters for the sea trial are provided in Table 1.

**Figure 13:**
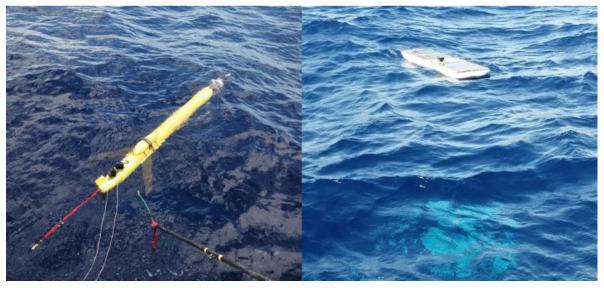
The equipment deployment for the sea trial.

The surface wave-energy glider traversed the water surface by harnessing wave energy, while the underwater glider followed a profiling trajectory underwater at a speed of approximately 1 knot. The maximum diving depth of the underwater glider was limited to 850 m. The equipment deployment for the sea trial experiment is shown in Figure 13.

**Figure 14:**
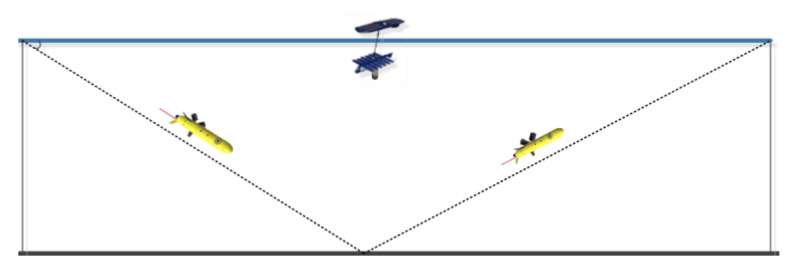
The relative movements of the glider.

During the experiment, the shore-based control center periodically sends communication requests to the surface wave-energy glider via satellite. The surface wave-energy glider, in turn, communicates with the underwater glider based on these requests and transmits the glider’s status information back to the shore-based control center through acoustic communication and satellite links. The control center then counts the received data packets and calculates the maximum communication distance each glider achieves. The relative motion of the glider during the trials is illustrated in Figure 14.

The trial data were processed, and the time-frequency domain oscillogram was recorded. This oscillogram was analyzed to evaluate the underwater glider’s communication performance at various depths and slant ranges. The specific working conditions of the glider’s communication are summarized in Table 2.

**Figure 15:**
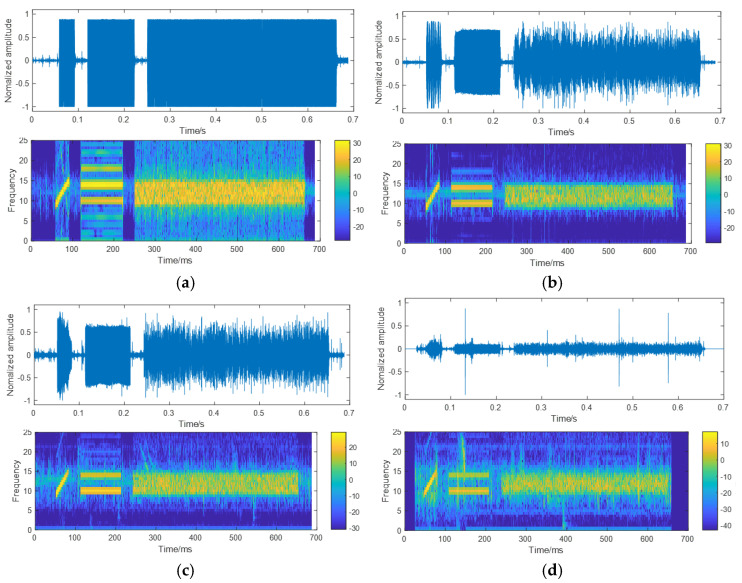
The primary time-frequency domain: (**a**) condition 1; (**b**) condition 2; (**c**) condition 3; (**d**) condition 4.

Figure 15 depicts the oscillogram of the time-frequency domain. Under working conditions 1 and 2, as shown in Figure 15a,b, the transmission distance is relatively short, resulting in high received power and full frame reception. A clear data reception frame structure is observed in the time-frequency domain, which does not adversely affect normal communication decoding. In contrast, Figure 15c,d correspond to working conditions 3 and 4, respectively. As the communication distance increases, the amplitude of the received signal decreases, leading to a blurred structure in the frequency-domain data reception frame.

In summary, acoustic gliders equipped with underwater acoustic communication modems are capable of achieving a maximum communication distance of 5 km.

## 5. Conclusions

This paper presents the design of an acoustic underwater glider equipped with underwater acoustic (UWA) communication capabilities for data observation and real-time transmission. The design effectively addresses challenges such as limited operational range and the inability of a single glider to provide real-time data. Including dual UWA transducers ensures a consistent energy transmission range and angle, even during posture changes of the underwater glider. Furthermore, implementing multi-carrier frequency shift keying technology enhances communication stability for UWA communication.

Sea trials conducted with the acoustic underwater glider demonstrated its ability to maintain communication over distances exceeding 5 km. In addition, the experiments in this study were limited to point-to-point communication and did not involve large-scale networking applications. In the future, when multiple gliders are networked for communication, it will be necessary to redesign the network protocols to match the new requirements. The findings of this study serve as a foundational step toward enabling inter-medium communication among heterogeneous underwater uncrewed vehicles, as well as between underwater, surface, space-based, and shore-based platforms.

## Figures and Tables

**Table 1:** UWA CP-MFSK settings.

Bandwidth	B	6 kHz
Cyclic-prefix length	$T_{cp}$	20 ms
No.data subcarriers	N	180
Blocks in one frame	M	9

**Table 2:** Details of the glider communication conditions.

Working Condition	Communication Depth of Glider/m	Communication Slant Range/m	Corresponding Time-Frequency Diagram
Working Condition 1	16	216	Figure 15a
Working Condition 2	660	1102	Figure 15b
Working Condition 3	315	2652	Figure 15c
Working Condition 4	511	5113	Figure 15d

## Data Availability

Data available on request due to restrictions (e.g., privacy, legal or ethical reasons).
